# Monitoring of plasma butyrylcholinesterase activity and hematological parameters in pesticide sprayers

**DOI:** 10.4103/0019-5278.40813

**Published:** 2008-04

**Authors:** S. K. Rastogi, Vipul K. Singh, C. Kesavachandran, Jyoti, M. K. J. Siddiqui, N. Mathur, R. S. Bharti

**Affiliations:** Epidemiology Section, Industrial Toxicology Research Centre, PB No. 80, Lucknow - 226 001, India; 1 Analytical Toxicology Section, Industrial Toxicology Research Centre, PB No. 80, Lucknow - 226 001, India

**Keywords:** Hematological parameters, pesticide sprayers, plasma butyrylcholinesterase3

## Abstract

To evaluate the health impact of spraying organophosphorus insecticides (OPs), 34 male sprayers in the mango belt of Malihabad, a small town located 27 km from Lucknow in North India was selected. Plasma butyryl cholinesterase (PBChE) and complete blood count were assessed among sprayers after spraying pesticides and the findings obtained were compared with those determined in a reference group (*n* = 18). The most common symptoms observed were burning sensation in the eyes (8.82%), itching/skin irritation (23.52%) and chest symptoms (32.35%) in the exposed workers. Plasma butyrylcholinesterase (PBChE) was significantly decreased in workers. The results indicated significant decrease in the mean value of hemoglobin, hematocrit and platelets count; however, significantly higher count of leukocytes was also observed in the exposed group (sprayers) compared to that observed in the control group (*P* < 0.05). Monitoring of PBChE in pesticide sprayers could be useful to predict and prevent health hazards of OPs.

## INTRODUCTION

Organophosphorus insecticides (OPs) are frequently used and sprayed in mango plantation of Malihabad (Lucknow, U.P.) which is a mango belt in north India and remain an important source of poisoning. Most occupational exposures to these pesticides occur from skin absorption and inhalation as the sprayers are from poor family and not in a habit to use face masks. There are strong associations between exposures to aerial pesticides and symptoms and cholinesterase activity is significantly reduced in the exposed pesticide sprayers.[1–3] Cases of chronic neurotoxicity among farm workers exposed to organophosphorus insecticides have been reported in several literature.[4–9] Most OP insecticides exert their toxicity in target and non target organs through inhibition of acetylcholinesterase in nerve and muscle tissue.[10–12] Actual exposures to insecticides can be assessed by biological monitoring of human tissues and body fluids.[13] The most economical blood test for the monitoring of farm workers who are exposed to organophosphorus insecticide is Plasma butyrylcholinesterase activity, its inhibition is taken as a biomarker for exposure.[14–15] Monitoring of Plasma butyrylcholinesterase (PBChE) has been recommended in the OP exposed population as this could be a useful biomarker to predict and prevent health hazards of pesticides.[13]

This study was therefore carried out to monitor the levels of Plasma butyryl cholinesterase and hematological parameters at the end of spraying day among the sprayers who use OP pesticides as their activities are significantly inhibited which is taken as a biomarker for OP exposure and compared with the unexposed population of the same age group from the similar region.

## MATERIALS AND METHODS

### About the pesticide sprayers

Thirty four pesticide applicators ranging in age from 20-25 years were selected for the study. Smokers and alcoholics were excluded from the study. Pesticide like monocrotophos, phosphomidon, dichlorvos, malathion, endosulfan, thiodon, methyl parathion, dimethoate and carbaryl were used by applicators. Clothing and body contact with spray solution were visually observed. The spray procedures and condition of the equipments, eating and drinking habits and personal cleanness were also observed and recorded. Mixing of all the pesticides with bare hands, leakage from tanks of pesticide and taking food without proper washing during and after spraying operations were found to be very common among all pesticide applicators.

### Health studies

A complete clinical examination of the workers including general and physical examination of nervous, respiratory, cardio vascular, gastro intestinal, ocular, skin and musculo-skeletal systems were conducted for each sprayer. Consent was taken from the worker to carry out the studies and the result of the study was reported back to each individual along with the suggestions for precautionary measures. A control group of 18 subjects were selected for the study from the same locality with similar age group, socio economic status and not exposed to pesticides.

### Biochemical parameter

Plasma butyrylcholinesterase (BChE) activity was determined by the method Ellman *et al*.[16] and as modified by Chambers and Chambers[17] by taking butyrylthiocholine iodide as substrate and expressed as m moles hydrolyzed/h/L Plasma.

### Hematology

To study the effect on hematological parameters viz, total red blood cell count, total leukocyte count, differential leukocyte count, hemoglobin percentage, hematocrit, mean corpuscular volume (MCV), mean corpuscles hemoglobin concentration (MCH) and platelet count were estimated on each workers and controls using standard methods in hematology.[18]

### Statistical analysis

Significance of mean values of biochemical parameters and hematology in pesticide sprayers and controls were compared using students ‘t’ test.

## RESULTS

Table 1 shows the physical characteristics of workers. Fifteen percent of subjects had experiences of one or more symptoms potentially associated with exposure to pesticides. Burning sensation in the eyes/face, itching and skin irritation, skin rash, dizziness, nausea etc were the most prevalent symptoms. Upon physical examination of sprayers performed at the beginning of the study, none of them displayed unequivocal cholinergic signs; however, the only prominent finding was an elevated incidence and prevalence of skin effects (23.52%) and they are consequences of direct irritative action on the epidermis or the result of allergic reactions and may be related with the lack of use of protective clothing found in this group. Burning sensation in the eyes (8.82%), burning signs in throat (8.82%), itching, rashes and burning sensation of face (17.64%), stomach cramps, epigastric and abdominal pain (17.64%), chest discomfort, chest tightness, productive cough, dyspnea, basal crepitation of both lungs and dry cough (26.42%) were observed in sprayers. Control subjects were reported NAD (No abnormality detected).

**Table 1:** Comparison of physical characteristics between the pesticide sprayers and controls

Variables	Controls (*n* = 18)	Sprayers (*n* = 34)
	Mean ± SD	Mean ± SD
Age (Y)	25.25 ± 3.06 (21-33)	26.93 ± 8.27 (14-50)
Height (cm)	170 ± 6 (162-180)	159 ± 5 (148-167)
Weight (kg)	62.75 ± 10.24 (46-81)	49.9 ± 5.85 (43-68)

Parenthesis indicates the range

Blood samples were collected from pesticide sprayers following spraying. Complete blood count (CBC) was determined with an automated blood analyzer. The results indicated significant decrease in the mean value of hemoglobin in the exposed group compared to that observed in the control group possibly as a result of the decreased size of red blood cells or impairment of biosynthesis of heme in bone marrow[19,20] [Table 2]. Another possible interpretation is binding of organophosphorus insecticides on iron, followed by a lack of incorporation of iron in hemoglobin.[21] In addition, the significant decrease in hematocrit is in agreement with that reported for Spanish green house sprayers.[22] However, the significantly higher leukocytes and reduced platelets count observed in the sprayers signifies the activation of the defense mechanism and immune system, which could be a positive response for survival.[23] Some of the previous studies have also shown leucocytosis following exposures to insecticides, including organophosphates.[24,25]

**Table 2:** Complete blood counts of the pesticide sprayers and controls

Hematological profile	Exposed sprayers (*n* = 18)	Controls (*n* = 34)
	Mean ± SD	Mean ± SD
Red blood cell count	4.29 ± 0.14	4.22 ± 0.11
Leukocyte count (10^3^ cells/)	7.57 ± 0.21	6.33 ± 0.27
Hemoglobin	12.86 ± 0.13	13.56 ± 0.18*
Hematocrit	44.64 ± 0.67	45.69 ± 0.55*
MCV	80.45 ± 1.01	82.44 ± 0.85
MCH	26.62 ± 0.44	28.21 ± 0.86
MCHC (g/dl)	31.69 ± 0.20	32.35 ± 0.19
Platelet count (10^3^ cells/)	200.5 ± 16.80	239.64 ± 12.68*

MCV - mean corpuscular volume; MCH - mean corpuscles hemoglobin concentration

* Statistically significant (*P* < 0.05)

**Figure 1:**
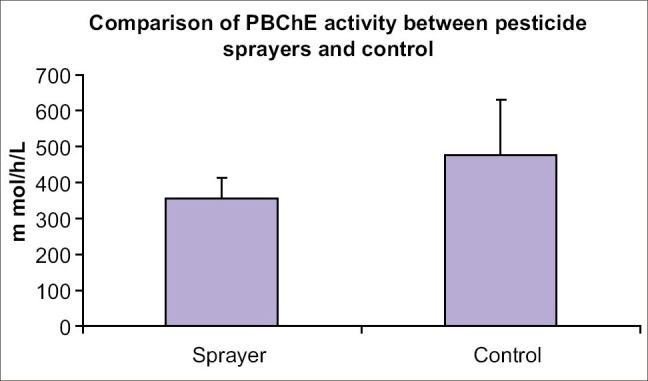
Biochemical profile in the blood of pesticide sprayers (*n* = 18) and controls (*n* = 34)

[Figure 1] shows, the activity of Plasma BChE enzyme were significantly lower in sprayers as compared to the controls (*P* < 0.05). Hematological parameters were all in normal range among control subjects.

## DISCUSSION

In undertaking this kind of study, we must first consider the difficulty of quantification and assessment of the effects of the exposure that may lead to chronic intoxication. Multiple exposures of different pesticides which might interact in an additive or multiplicative way and so could affect the pattern of health effects expected in the case of mono-exposures. The other important points that have also affected this study include the large number of spraying made and the usage of various pesticides during the period of study, often in combinations, some commercially available but the majority made by the sprayers themselves. Despite all these limitations, it is possible to draw conclusions about the overall health risks of these complex exposures.

The findings suggest that chronic exposure to different pesticides in a bizarre fashion suggests the involvement of many different organ systems, including skin, digestive and respiratory systems. However it is difficult to gauze the effects on the health of workers of combined exposure to different pesticides because toxic manifestations may not be specific for each toxicant. During the 1960s, however, it became evident that persistent pesticides were having an adverse impact on ecologic communities.[26] This led to a number of more extensive epidemiologic investigations exploring the possible impact of these exposures on human health. These studies faced numerous methodological problems common to environmental epidemiology and even today, our understanding of the relationship between pesticides and human health is limited.[27] That is the reason why it would be necessary to distinguish which signs and symptoms are due to chronic exposure to pesticides and which are not. Nevertheless, when using various types of pesticides in different doses and in different times of exposure, it is difficult to assess and quantify the risk of exposure.

In contrast to previous studies that reported changes in the lymphocyte and monocyte counts[20,23–25] we found no leucocytosis in our study subjects. Leucocytosis has been recorded following exposures to insecticides including organophosphates. We also could not observe decrease in hemoglobin as reported in a previous report.[13] The biochemical parameter Plasma butyrylcholinesterase activities were significantly decreased in the sprayers at the end of work day thereby suggesting acute poisoning from multi exposure to different pesticides. Decline in Plasma butyrylcholinesterase supports earlier findings of Hillman (1994), Misra et al (1994), Lopez-Carillo et al (1993) and Clarke *et al*. (1997).[14,15,28,29]

## CONCLUSION

Plasma butyrylcholinesterase (PBChE) were significantly inhibited due to multiple exposure to different pesticides. Mixing of pesticides before spraying and direct re entry soon after spraying play significant roles in reducing Plasma butyrylcholinesterase activity. Blood count done on the sprayers failed to demonstrate leucocytosis and decrease in hemoglobin levels among the sprayers. Plasma butyrylcholinesterase (PBChE) is recommended as a biomarker for exposure to OP pesticides. The observed significant decrease of PBChE following pesticide spraying in the exposed group coupled with changes in hematological profile recommend monitoring of PBChE and complete blood count (CBC) as it is useful to predict and prevent health hazards of OP pesticides. Finally it would be necessary to undertake long-term studies on pesticide sprayers because of the lack of a homogenous pattern of exposure.
